# Surgical treatment of innominate artery and aortic aneurysm: a case report and review of the literature

**DOI:** 10.1186/1749-8090-8-141

**Published:** 2013-06-01

**Authors:** Erdinc Soylu, Leanne Harling, Hutan Ashrafian, Vania Anagnostakou, Dimitris Tassopoulos, Christos Charitos, John Kokotsakis, Thanos Athanasiou

**Affiliations:** 1 Department of Surgery and Cancer, Imperial College London, London, UK; 2 Second Department of Cardiac Surgery, Evangelismos General Hospital Athens, Athens, Greece; 3 Department of Surgery and Cancer, Imperial College London, 10th floor QEQM Building, St. Mary’s Hospital, Praed Street, London, W2 1NY, UK

**Keywords:** Brachiocephalic trunk, Aneurysm, Surgery

## Abstract

Innominate artery (IA) aneurysms represent 3% of all arterial aneurysms. Due to the risk of thromboembolic complications and spontaneous rupture, surgical repair is usually recommended on an early elective basis. We present the case of 81-year-old Caucasian male presenting with atypical anterior chest pain secondary to a large innominate artery aneurysm who underwent successful open surgical repair at our institution. In our experience, open correction via median sternotomy with extension into the right neck provides excellent exposure and facilitates rapid reconstruction with good short and long-term outcomes. Minimally invasive and endovascular approaches provide emerging alternatives to open IA aneurysm repair, however further research is required to better define optimal patient selection criteria and determine the long-term outcomes of these novel therapies.

## Background

Aneurysms of innominate artery (IA) make up approximately 3% of all arterial [1] and 3% of all supra-aortic vessel aneurysms [2]. The majority of IA aneurysms are atherosclerotic in aetiology [3], however alternative causes include syphilis, tuberculosis, Kawasaki’s disease, Takayasu’s arteritis, Behçets disease, connective tissue disorders and angiosarcoma.

Aneurysms of the IA commonly present secondary to local compression, thrombosis or distal embolization, giving rise to a diverse range of symptoms including dyspnoea, dysphagia, hoarseness, facial and upper limb oedema, chest pain, digital ischaemia, right hemispheric symptoms, amorosis fugax, vertebrobasilar syndrome, and rarely the presence of a pulsatile anterior thoracic mass [4].

Untreated IA aneurysms are liable to rupture, compress nearby viscera or give rise to cerebrovascular embolism [3]. Rupture is the most threatening presentation occurring in approximately 11% of all patients [5]. The risk of rupture is however more likely following trauma or in patients with underlying connective tissue disease [5,6], and early repair by means of open surgery or endovascular therapy is therefore advocated in patients with symptomatic disease, associated aortic arch aneurysms, saccular aneurysms or isolated asymptomatic aneurysms of >3cm diameter [5].

Over the recent years, there has been an increase in the number of reports of endovascular innominate aneurysm repair however the majority of cases are still treated using an open surgical technique via median sternotomy with or without extension to right neck (Table 1).

**Table 1:** Summary of observed short and long-term complications

**Access route**					**Short-term outcomes (<30 days)**			**Long-term outcomes (>30 days)**		
	**Study**	**Year**	**Elective: Emergency**	**Length of stay**	**Mortality**	**Morbidity**	**Graft patency**	**Morbidity**	**Graft patency**	**Follow-up**
**Endovascular**										
	Angiletta [12]	2012	1:0	-	0	-	-	-	Patent	Alive and developed an aneurysm of the right carotid artery bifurcation at 96 months
	Taha [15]	2010	1:0	-	-	-	Patent	-	Patent	-
	Puech-Leao [16]	2001	1:0	7	0	-	-	-	Patent	Alive and symptom free at 24 months
	Park [17]	2001	1:0	-	-	-	Patent	-	Patent	-
**Hybrid endovascular and sternotomy**										
	Mellisano [13]	2004	1:0	8	0	Atrial fibrillation, cardiac arrest, pulmonary effusion	Patent	-	-	Alive and symptom free at 3 months
**Cervical approach**										
	Takach [18]	2007	1:0	-	0	-	-	-	-	-
**Partial median sternotomy**										
	MacLean [19]	2007	1:0	-	-	-	-	-	-	-
	Mori [20]	2004	1:0	-	-	-	-	-	-	-
	Lane [21]	1951	1:0	-	0	Phrenic nerve damage, fever	-	Muscle atrophy/weakness	-	-
**Full median sternotomy**										
	Constenla [11]	2012	1:0	-	0	Respiratory failure, pneumonia	Patent	-	-	Alive and symptom free at 12 months
	Oswal [22]	2011	1:0	-	0	-	-	-	-	Alive and symptom free at 12 months
	Lu [14]	2011	1:0	-	0	-	-	-	-	Alive at 20 months
	Yuan [23]	2009	1:0	-	-	Stroke	-	-	-	-
	Da Col [24]	2007	1:0	12	-	-	Patent	-	-	-
	Oruganti [25]	2006	1:0	-	-	-	Patent	-	-	-
	Saito [26]	2005	1:0	-	-	-	-	-	-	-
	Kieffer [5]	2001	23:4	-	3	Neurological deficits (2), dysphonia (3), haemorrhage (1), artficial ventilation (5), multiple organ failure (2)	Patent (21), unknown (3)	Dysphonia (1), mediastinitis (1)	Patent (14), unknown (3)	7 deaths (cancer=3, cardiac=2, accident =1, unknown=1) during 85 months (3–194)
	Chiappini [27]	2001	1:0	-	0	-	-	-	Patent	Alive at 6 months
	Guibaud [28]	2001	0:1	-	0	-	-	Subclavian steal syndrome	-	-
	Najafi [29]	1999	1:0	6	-	-	-	-	-	-
	Villegas-Cabello [30]	1996	1:0	-	-	-	-	-	-	-
	Adkins [31]	1993	1:0	20	-	-	-	-	-	-
	Bower [3]	1991	6:0	-	0	Atelectasis (1), pneumonitis (1), brain stem infarct (1)	-	Stroke (1)	-	3 late deaths (cancer=1, stroke=1, vasculitis and heart disease=1), lost to fallow up (1), unkown (1). 5 year survival=62.5%
	Thomas [32]	1972	1:0	-	1	Thromboembolism	-	-	-	-
**Combined full median sternotomy and cervical approach**										
	Ketonen [33]	1983	1:0	-	0	Wound haematoma of femoral incision	-	-	-	Alive and symptom free at 108 months
			1:0	-	0	-	-	-	Patent	Died due to the laryneal cancer at 2 months
	Murray [34]	1971	1:0	-	0	-	-	-	-	Alive and symptom free at 10 months
	Zintel [35]	1960	1:0	14	0	Right arm ischaemia, wound infection	-	-	-	Alive and no change in pre-operative neurological deficit at 36 months
**Full median sternotomy with anterior neck dissection**										
	Ikonomidis [36]	2004	1:0	7	0	Persistent arm swelling	Patent	-	Patent	Alive and symptom free at 12 months
	Takaba [37]	2003	1:0	20	-	-	-	-	-	-
	Kasashima [38]	2000	1:0	34	0	Right hemianopia	-	-	-	Alive, right upper quadrantic hemianopia and right facial anhidrosis on day 34
	Tominage [7]	1988	-	-	-	-	-	-	-	-
	Schumacher [39]	1979	1:0	14	0	Respiratory dysfunction	-	-	-	Alive, right vocal paralysis and hoarseness persisted, dysphagia relieved at 9 months
	Cook [40]	1960	1:0	-	1	-	-	-	-	-
			1:0	70	0	Infection	-	Paresis, haemorrhage, reintervention	-	-
	Kirby [8]	1953	1:0	17	0	Paralysis of left arm	-	Paresis	-	-
**Thoracotomy**										
	Cook [40]	1960	1:0	-	0	-	-	-	-	Alive and symptom free at 55 months. Imrovement of aneurysm
**Conservative**										
	Yang [41]	2009	1:0	-	-	-	-	-	-	-
	Butman [42]	1983	1:0	-	1	Brain stem/cerebellar infarct	-	-	-	-
**Unspecified**										
	Gordon-Taylor [43]	1950	-	-	19	Hemiplegia (2), cerebral symptoms (2), haemorrhage (4), uraemia (1), Respiratory dysfunction/failure (2), pneumonia (1), mediastinitis (1), infection (2), reintervention (1)	Obstructed=1	Paresis (1), cardiac breakdown (1), haemorrhage (2), persistent pulsation (1), rupture of aneurysm (1), ligature cut through aorta (1), pneumonia (1), nephritis (1), recurrence (2)	-	10 deaths

We present a case of IA aneurysm involving the aortic arch treated via an open approach, and review the current literature on the role of open surgical and minimally invasive interventions for the treatment of IA aneurysms.

## Case presentation

**Figure 1:**
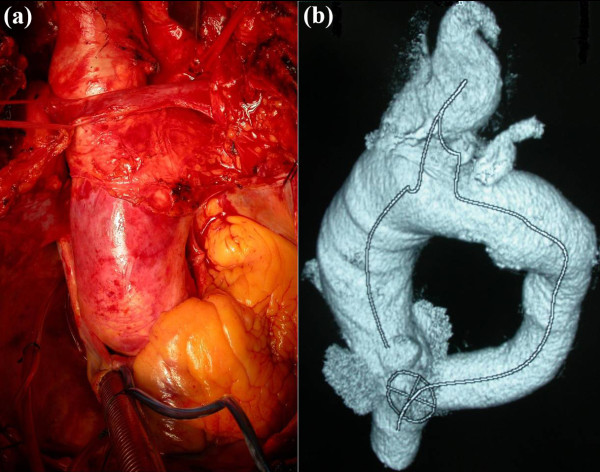
(a) Intra-operative finding of a large IA aneurysm extending into the aortic arch (b) Pre-operative 3D CT reconstruction of the aneurysmal segment.

A 81- year- old Caucasian male with hypertension was admitted to our hospital with atypical anterior chest pain. History and physical examination were unremarkable. However, transthoracic echocardiography demonstrated an aneurysm of the ascending aorta (5.4cm) with mild aortic regurgitation and an LVEF of 50%. Computerized tomographic angiogram visualized a degenerative aneurysm of the ascending aorta (5.5cm), proximal arch (4.2cm) and innominate artery (4.6cm) (Figure 1).

Under general anaesthesia, median sternotomy was performed extended into the right neck along the medial edge of the sternocleidomastoid. A second left subclavicular incision was made to expose the left axillary artery. Following systemic heparinization, cardiopulmonary bypass (CPB) was instituted with an arterial cannula introduced in the left axillary artery and a two-stage venous cannula introduced into the right atrium, maintaining a flow rate between 2.2 and 2.4 L.min^-1^.m^-2^_._ Upon cardiac fibrillation, the ascending aorta was cross-clamped and resected above the coronary ostia at the level of the sinotubular junction. Myocardial arrest was achieved using cold crystalloid cardioplegia (25 ml.kg^-1^).

A four-limbed (28 × 10 × 8 × 8 × 10mm) Dacron aortic arch graft was prepared and anastomosed proximally to the aortic sinotubular junction with external Teflon strip reinforcement. At a target bladder temperature of 26°C, the patient was placed in the Trendelenburg position; CPB flow arrested, and the residual ascending aorta and proximal arch were resected preserving the ostia of the IA, left common carotid (LCCA) and left subclavian (LSA) arteries. The aneurysmatic IA was completely resected from its origin in the arch until its distal bifurcation to the right subclavian (RSA) and right common carotid (RCCA) arteries. Unilateral antegrade selective cerebral perfusion (ASCP = 10ml/kg/min, maintenance radial pressure 50mmHg) was instituted via a cerebral perfusion catheter to the LCCA and placing soft clamps to the RSA, RCCA and LSA. Cerebral monitoring was achieved by means of transcutaneous cerebral oximetry and electroencephalogram.

**Figure 2:**
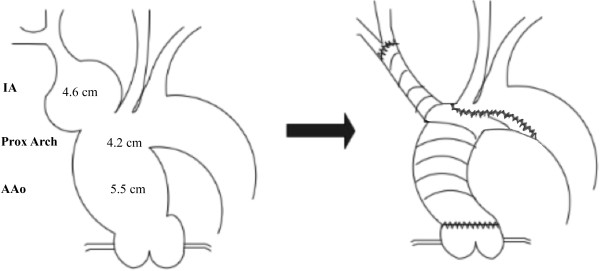
Schematic demonstrating pre-operative anatomy and surgical repair.

**Figure 3:**
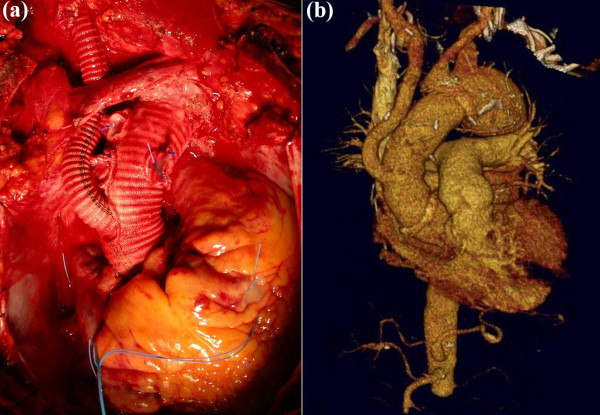
Post operative images demonstrating (a) Final open surgical repair (b) 3D CT reconstruction demonstrating in situ graft reconstruction.

The Dacron graft was trimmed and the two distal side limbs (8mm) ligated. The distal end was anastomosed in a hemiarch fashion to the aortic arch proximal to the origin of LCCA and reinforced with external Teflon strips. The side branch of the graft was then anastomosed in an end-to-end fashion to the distal IA. After systematic de-airing, the distal anastomosis to the arch was completed, and systemic perfusion and rewarming commenced (Figures 2 and 3).

The CPB time was 110 minutes, ASCP time 45 minutes, lower body circulatory arrest time 38 minutes and myocardial ischemic time 85 minutes. The patient was extubated 12 hours after surgery with a total ITU stay of 2 days. The post-operative course was unremarkable. Repeat CT three months after surgery and showed an excellent result.

## Conclusions

Since its first description by Valentine Mott in 1818, significant advances have been made in the surgical repair of innominate artery aneurysms. Most attempts in 19th and the beginning of 20th century were limited to ligation of the aneurysm, resulting in mortality rates as high as 30–78% [5]. Although the method described by Kimura in 1908 involving triple ligation and excision of the aneurysm was technically successful [35], cerebral injury following the ligation of the right common carotid was a serious complication. It was not until 1952, when Kirby and Johnson [38] had reported successful resection of IA aneurysm and reconstruction of circulation to RCCA by end-to-end anastomosis. Utilization of bifurcated aortic homograft by DeBakey and Crawford in 1956 [42] and introduction of the bifurcated Dacron graft by Hejhal et al. [43] in 1965 were milestones in reducing the mortality to recently observed levels of 5% [3]. However, operative mortality rate of emergency operations are still being reported as 50% [3], emphasising the need for early elective repair where possible.

Today the commonest surgical approaches to the IA include median hemisternotomy combined with right anterior thoracotomy in the 3rd intercostal space and the right supraclavicular fossa, median sternotomy with extension along the medial border the right sternocleidomastoid muscle and the more recently reported cervical approach (Table 1).

Although employed less frequently than open surgical repair, endovascular, minimal access cervical or hemisternotomy techniques have more recently been associated with fewer short-term complications, shorter hospital stay, comparable graft patency and similar short- and long- term mortality to open surgery (Table 1). However despite these potential benefits, careful patient selection is needed and long-term outcome data remains lacking. Endovascular treatments can also be challenging in cases of bovine arch morphology [16], where the aneurysmal neck is inadequate for attachment of the graft or when the distal innominate artery is involved [7,11]. Furthermore, covered endovascular stents may require long-term antiplatelet therapy and a closed approach presents diagnostic difficulty in ruling out any malignant processes underlying aneurysm formation [18].

This case demonstrates the safe application of median sternotomy with extension to the right anterior neck to perform open repair of a large, complex aneurysm of the IA involving the aortic arch. Although open repair carries a relatively higher short-term post-operative morbidity (Table 1), improvement in surgical techniques over the course of the last century has facilitated excellent long-term results. However, the increasing use of minimally invasive strategies presents an emerging alternative in the surgical treatment of these patients. The heterogeneous nature of multiple small studies with a lack of large multi-centre studies and late follow up however limits the data currently available. Further research is therefore required to assess both the long-term patency and mortality associated with minimally invasive surgical approaches.

## Consent

Written informed consent was obtained from the patient for publication of this Case report and any accompanying images. A copy of the written consent is available for review by the Editor-in-Chief of this journal.

## Abbreviations

IA: Innominate artery; CPB: Cardiopulmonary bypass; LCCA: Left common carotid artery; LSA: Left subclavian artery; RSA: Right subclavian artery; RCCA: Right common carotid artery; ASCP: Antegrade selective cerebral perfusion.

## Competing interests

There are no financial or non-financial competing interests.

## Authors’ contributions

JK, VA, DT and CC carried out the surgical procedure, participated in provision of clinical information and reviewed the manuscript. ES and LH performed the literature review and drafted the manuscript with supervision and assistance from HA and TA. LH, HA and TA finalized the manuscript. All authors read and approved the final manuscript.
